# Thoracic circumference as a predictor of metabolic syndrome and changes in its components in non-obese adults

**DOI:** 10.1186/1758-5996-7-S1-A156

**Published:** 2015-11-11

**Authors:** Ana Paula Abreu Martins Sales, Nadia Tavares Soares, Maria Helane Costa Gurgel Castelo, Clarisse Mourão Melo Ponte, Virginia Oliveira Fernandes, Ana Paula Dias Rangel Montenegro, Renan Magalhães Montenegro

**Affiliations:** 1 Universidade Federal do Ceará, Fortaleza, Brazil

## Background

The use of anthropometric indices to screen for metabolic and cardiovascular risk factors has been the focus of many studies in recent yrs. Besides waist, abdominal and pelvic circumferences, the upper body segment circumferences have been the most investigated, particularly cervical circumference. However, few studies have evaluated thoracic circumference (TC) for this purpose.

## Objective

This study aims to evaluate the relations among TC with the components of metabolic syndrome (MetS) and the ability of this anthropometric parameter in identify MetS among adults with a body mass index (BMI) between 18.5 and 29.9 kg/m^2^.

## Materials and methods

Correlations among TC with the components of MetS have been analyzed with correlations tests (Pearson or Spearman). The ability of the parameter regarding the identification of MetS has been analyzed using ROC curve.

## Results

There were evaluated 85 men and 191 women and mean age was 34.9 ±11.2 yrs. (33,7 yrs. men; 35,5 yrs. women). The group BMI average was 25.0±2.9 Kg/m2 (25.0±2,84 men; 24,9±2,86 women); waist circumference average was 86,9±8,2 cm (89,6±7,9cm men; 85,6±8,1cm women); TC average was 90.0±6,7 cm ( 85,8±6,1 men; 87,5±5,2cm women). The prevalence of metabolic syndrome in this group was 28%. TC was found to correlate with all components of MetS except glycaemia, being these correlations stronger with waist circumference. When using ROC curve TC was able to identifying MetS, with best Results in women. TC values of 95.8 cm and 87.3 cm respectively for men and women have presented the greater sensitivity for the prediction of MetS, with specificity ≥ 50%.

## Conclusion

These findings suggest that thoracic circumference represents a promising option for metabolic and cardiovascular risk evaluation because this measurement is simple to obtain during clinical evaluation and may identify individuals at higher risk of developing MetS.

